# Develop a novel non‐coplanar trajectory optimization algorithm for an efficient 4pi spot‐scanning proton Arc (SPArc_‐4pi_) treatment delivery

**DOI:** 10.1002/mp.70639

**Published:** 2026-08-18

**Authors:** Xiaoda Cong, Shupeng Chen, Peilin Liu, Peng Chen, Xiangkun Xu, Xiaoqiang Li, Peter Chen, Prakash Chinnaiyan, Xuanfeng Ding

**Affiliations:** ^1^ Department of Radiation Oncology Corewell Health William Beaumont Hospital Royal Oak Michigan USA; ^2^ Department of Radiation Oncology Emory University Atlanta Georgia USA; ^3^ Department of Radiation Oncology Cleveland Clinic Cleveland Ohio USA

**Keywords:** 4pi arc, non‐coplanar proton arc, spot‐scanning arc therapy

## Abstract

**Background:**

The degree of freedom has been recognized as a key factor in particle beam therapy, such as improving the dose conformity and linear energy transfer distributions. Therefore, proton arc therapy has drawn significant interest in the Society of Radiation Oncology as a potentially efficient and optimal treatment option for cancer patients. However, state‐of‐the‐art spot‐scanning proton arc therapy (SPArc) still relies on single or dual‐ co‐planar arc trajectories, which limits its capacity to advance the treatment outcome further. There is an urgent need to explore particle beam therapy's full potential via 4pi.

**Purpose:**

This study aims to develop the first 4pi arc optimization algorithm that searches for efficient arc trajectories in 4pi space, explores the potential dosimetric improvements, and demonstrates its feasibility through simulation.

**Method:**

Dynamic Programming, originally from control theory, was translated into this new concept of the 4pi SPArc optimization algorithm (SPArc_‐4pi_) for the trajectory search and route decision‐making. It breaks down the complicated and high‐computational‐demand main problem into a series of small sub‐problems and searches for delivery‐efficient 4pi arc trajectories through an iterative approach. Five different disease sites, e.g., head & neck, partial brain, clival brain chordoma, lung and pancreatic cancer, were used for testing purposes. Conventional Intensity Modulated Proton Therapy (IMPT) and 2D co‐planar arc (SPArc_‐2d_) plans were generated as a benchmark. Treatment delivery efficiency was evaluated through a published and validated dynamic arc system controller. Plan quality was assessed through target coverage and organ at risk (OAR) sparing.

**Result:**

The new SPArc_‐4pi_ demonstrated superior dosimetric performance across various evaluation metrics for all five disease sites. The simulation result shows that SPArc_‐4pi_ is able to be delivered within a reasonable time of around 5–11 mins, which is comparable to the SPArc_‐2d_ plans

**Conclusion:**

The study introduced the first optimization algorithm for SPArc_‐4pi_ technique. It not only showed SPArc_‐4pi_ ‘s potential to improve the plan quality via a greater degree of freedom compared to the conventional IMPT and state‐of‐art 2D co‐planar SPArc‐2d technique but also demonstrated its feasibility for future clinical implementation based on the current proton beam therapy system's machine characteristics.

## 1 INTRODUCTION

Since the introduction of Spot‐scanning Proton Arc therapy (SPArc) in 2016,^1^ the importance of increasing the degree of freedom has been gradually realized in particle beam therapy.^2^ Compared to conventional Intensity‐Modulated Proton Therapy (IMPT) with limited beam angles, SPArc has demonstrated its superior dosimetric plan quality across a broad range of clinical indications, including breast,^3^ lung,^4^ brain,^5^ head and neck,^6^ and prostate cancers^7^ and potential enhancements in linear energy transfer (LET)^8^ distribution via arc trajectory in treatment planning optimization and delivery.^7^, ^8^ Through an industrial‐academic collaboration, a prototype was successfully established in a clinical proton therapy system at Beaumont Proton Center, Corewell Health, which marked a major milestone toward clinical implementation.^9^

However, most in silico SPArc investigations are still based on the limited 2D co‐planar arc trajectory without fully utilizing the degree of freedom from the 4pi spherical space. Meanwhile, there is still ongoing debate about whether or not the proton arc technique will bring a revolutionary change to our future radiation oncology community.^10^ Thus, the full potential of particle beam therapy remains to be explored beyond the 2D co‐planar arc trajectories.^11^ Based on recently developed technology, the dynamic SPArc therapy system,^1^ we decided to advance it further to the entire 4pi space, enabling simultaneous Patient Positioning System (PPS) and gantry rotation during the planning and delivery phases.

In the field of photon radiotherapy, the concept of 4pi has been widely clinically implemented for decades through various machines such as Cyberknife and gamma knife as these systems have demonstrated its superior dosimetric quality through numerous beam angles or radiation sources from a 4pi solid angle compared to the conventional 2D co‐planar Volumetric Modulated Arc Therapy (VMAT).^12^, ^13^, ^14^, ^15^ Its historical significance and clinical outcome have been a point of interest for researchers and clinicians to develop a more versatile radiotherapy system based on C‐arm LINACs, extending such benefits to more patient populations and disease sites.^16^, ^17^ Throughout the last decades, photon 4pi optimization algorithms and prototype delivery systems have been successfully developed in clinical settings, demonstrating their feasibility, safety, efficiency, and superior dosimetric plan quality.^18^, ^19^ However, such a 4pi concept on C‐arm LINACs might not be directly applied to proton beam therapy due to significant differences such as (1) the mechanical constraints of the gantry size: a couple of tons (LINACs) versus hundreds of tons (proton gantry) and (2) treatment delivery mechanisms: Multi‐leaf collimator versus energy layer and spot scanning. These additional factors and dimensions made it more challenging to explore and implement the 4pi concept in the field of particle beam therapy.

Spot‐scanning Proton Arc (SPArc) optimization through 2D co‐planar trajectory has been recognized as a mathematical challenge.^9^ Though numerous efforts have taken place to address treatment delivery efficiency and plan quality, there are still no golden solutions for SPArc optimization at this stage.^20^, ^21^, ^22^, ^23^, ^24^, ^25^, ^26^, ^27^, ^28^ Recently, the publication of SPArc therapy indicated that it is an NP‐hard problem.^29^ This means that evaluating the complete solution space and generating a solution will include extreme time costs even with state‐of‐the‐art computer systems,^30^ let alone expanding the trajectory search and optimization from a 2D co‐planar arc to a 4pi space.^31^ Therefore, the development of a new efficient optimization algorithm is required to advance the technique to a 4pi space and ensure it can be delivered within a reasonable treatment time.

Dynamic programming (DP) has been extensively studied in optimization algorithms within mathematics.^32^ The fundamental concept of DP involves breaking down complex problems into smaller, manageable subproblems and then constructing optimal solutions by recursively solving these subproblems.^33^ This approach significantly reduces computation time compared to other optimization algorithms.^34^ The principles of DP are applied in various optimizations and are foundational in solving Markov decision process.^35^ These unique features allow us to explore the feasibility of incorporating the DP into the 4pi Spot‐scanning Proton Arc therapy (SPArc_‐4pi_) optimization to solve a large and complex optimization problem.

In this study, we introduce a novel treatment planning and delivery technique advancing the particle beam therapy from 2D co‐planar arc (SPArc_‐2d_) to the 4pi space and address its clinical feasibility and potential dosimetric plan quality improvement through comprehensive in silico study and end‐to‐end treatment delivery simulation. This is the very first study to explore the feasibility of planning and delivering the particle beam therapy through the 4pi space.

## 2 METHOD AND MATERIALS

### 2.1 4pi optimization problem and trajectory search

**FIGURE 1:**
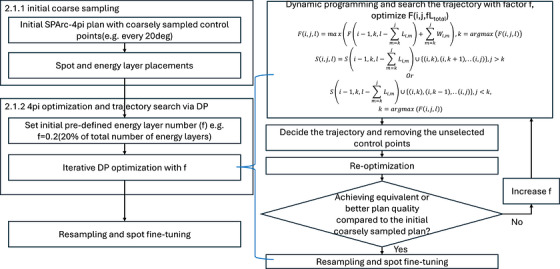
The design of the optimization and trajectory search for a SPArc_‐4pi_ plan.

The SPArc_‐4pi_ optimization and trajectory search is established through three main steps: (1) generation of initial coarse beam placement across the 4pi space; (2) trajectory search and route decision‐making through DP, in which it iteratively optimizes the total number of energy layers, and MU weighting of each control points via exploring every possible trajectory. (3) control point resampling process to increase the sampling frequency for a continuous and precise treatment delivery of the SPArc_‐4pi_ plan via a simultaneous PPS and gantry rotation. This novel concept offers extra freedom in optimizing and delivering the particle beam therapy treatment on top of the 2D co‐planar trajectory search. The study aims to demonstrate its feasibility by generating complicated SPArc_‐4pi_ plans with the current standard computation power and efficient treatment delivery with simultaneous and continuous PPS and gantry movement (Figure 1).

#### 2.1.1 Initial coarse sampling

The initial plan was generated through a coarsely sampled beam set with the interval of 20 deg for both PPS and gantry angles over the 4pi steradians. The IBA Protues®ONE room geometry and gantry design were applied with the gantry angle range from ‐40 to 180 deg and PPS rotation from 0 to 180 deg. Initial spot and energy layer placements were generated for each control point in a manner similar to the multi‐field IMPT planning approach.^36^

#### 2.1.2 4pi optimization and trajectory search via dynamic programming

Since the trajectory search in spherical space is an NP‐hard problem, the number of potential solutions has exponential growth based on the problem scale.^37^ However, if several hypotheses are made, the NP‐hard problem can be reduced to a solvable problem with polynomial computation time cost, and it can then be solved by DP optimization. Here we list our hypothesis and conditions based on the prototype DynamicARC® system's mechanical constraint and plan optimization algorithm design:

1. The initial energy layer, spot, and beam angle placement define the upper limits of data input and the computation cost of the optimization problem. For example, the initial plan with a coarse sampling frequency of 20 degrees contains a hundred thousand spots and thousands of energy layers. No more new beam angles, energy layers, or spots will be added during the SPArc_‐4pi_ optimization process.
2. We hypothesize that the total number of energy layers and their delivery sequence is a reasonable surrogate and a representation of the total treatment delivery time in the pencil beam scanning (PBS) technique.^20^, ^21^, ^22^, ^25^, ^38^, ^39^, ^40^ In other words, searching the trajectory along a smaller total number of energy layers leads to a shorter treatment delivery time for SPArc_‐4pi_ while optimizing plan quality.
3. We hypothesize that the MU weighting of each control point reflects the contribution to the plan quality during the optimization. Filtering or removing the low MU weighting control point may not significantly alter the plan quality.^1^ In other words, selecting a trajectory along the higher MU weighting control points is normally considered as a better option than selecting a trajectory along the lower MU weighting control points in terms of plan quality.
4. We hypothesize that it is easier for a particle therapy system to accelerate, decelerate, and rotate PPS back and forth while the gantry will keep slowly moving clockwise or counterclockwise in one direction only. Because the hundreds of tons of proton gantry have huge inertia, there is a significant mechanical constraint to rotate the gantry back and forth rapidly compared to the PPS. Thus, in this study, the SPArc_‐4pi_ trajectory search algorithm allows the PPS to rotate freely while the gantry moves only in one direction from the beginning (‐40 degrees) to the end (180 degrees) in a clockwise direction (IBA Proteus®ONE gantry range).

**FIGURE 2:**
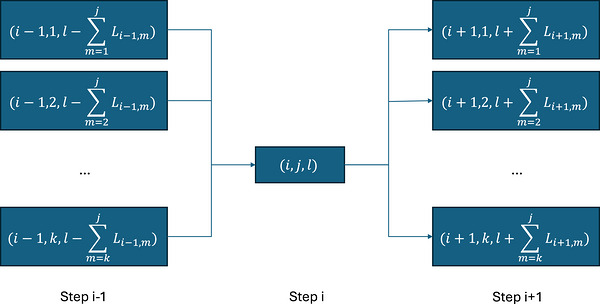
The state transfer between adjacent steps. The left part represents all the solutions from the previous i‐1 step, and the right part represents all the potential solutions for the next i+1 step. The center cell represents the decisions at the current i step: selecting a path with optimized MU weighting for each status of gantry position i, PPS position j and energy layer number l, according to equation (1). For each node k in i‐1 step, the energy layer number l should be subtracted with the sum of energy layers to be included in case the corresponding path is chosen, which is 𝑙−σ𝑚 = 𝑘𝑗𝐿𝑖−1,𝑚, to ensure the energy layer number does not exceed l at step i. This applies similarly to step i+1.

Based on these hypotheses and prior knowledge, we introduce the DP algorithm to search for a fast trajectory with high MU‐weighted control points. As we assume the gantry can only move forward, the gantry position among the control points can be considered as a step. Given the gantry position as i, PPS position as j, the total sum for the energy layer number of previously selected control points as l, then the current state of the system can be uniquely defined as (i,j,l). Assume in step i+1, the PPS position is k, the movement from (i,j,l) to $\left(i+1,k,l^{\prime }\right)$ is to choose control points (i+1,j)∼(i+1,k) adding to state (i,j,l). This will introduce an additional energy layer number $l^{\prime }-l=\sum_{m=k}^{j}L_{i+1,m}$ and MU weighting $\sum_{m=k}^{j}W_{i+1,m}$, in which $\left(L_{i,j},W_{i,j}\right)$ stands for the corresponding energy layer number and MU weighting for each initial control point (i,j). The algorithm is to find out the maximum MU weighting under every certain sum of energy layer numbers for (i,j,l), thus a path from (0,0,0) to (i,j,l), with MU weighting per energy layer efficiency optimized, can be found. A detailed explanation of this state transition is shown in Figure 2.

More specifically, we can write the path decision‐making function (a.k.a. state transition function) as follows:

(1) $$\begin{array}{cc} & F\left(i,j,l\right)=max\left(F\left(i-1,k,l-\sum_{m=k}^{j}L_{i,m}\right)+\sum_{m=k}^{j}W_{i,m}\right),\\ & k=argmax\left(F\left(i,j,l\right)\right) \end{array}$$

F(i,j,l) stands for the sum of MU weighting for all selected control points along the path. This function will ensure the path to every state (i,j,l) is with the optimized sum of MU weighting from a previous state $\left(i-1,k,l-\sum_{m=k}^{j}L_{i,m}\right)$, and the previous state also has a corresponding optimized path, recursively reaching the starting point (0,0,0).

Therefore, the trajectory search result or a set of selected control points, S(i,j,l), *can be defined* as follows:

(2) $$\begin{array}{cc} & S\left(i,j,l\right)=S\left(i-1,k,l-\sum_{m=k}^{j}L_{i,m}\right)\cup \\ & \left\{\left(i,k\right),\left(i,k+1\right),..\left(i,j\right)\right\},j>k \end{array}$$

**FIGURE 3:**
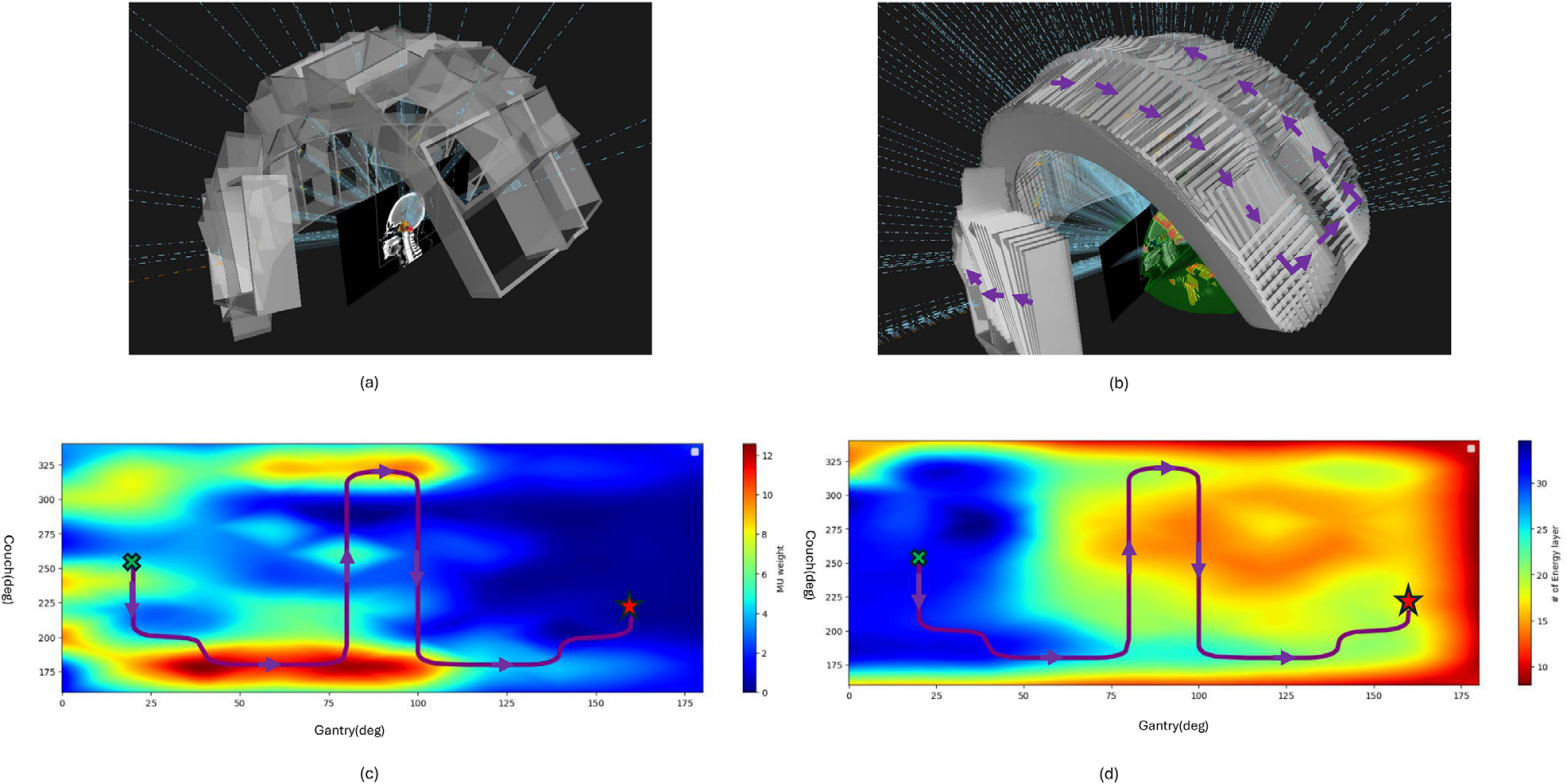
Trajectory search result of DP‐optimization after resampled for chordoma cancer as an example. (a) is initial control point placement, (b) is resampled SPArc‐4pi control point placement, (c) is the heatmap of weighting for initial control points, (d) is the heatmap of energy layer number for initial control points. The purple line is SPArc_‐4pi_ gantry and couch track.

Or

$$S\left(i-1,k,l-\sum_{m=k}^{j}L_{i,m}\right)\cup \left\{\left(i,k\right),\left(i,k-1\right),..\left(i,j\right)\right\},j<k,$$

$$k=argmax\left(F\left(i,j,l\right)\right)$$

j>k stands for clockwise PPS rotation and j<k stands for counterclockwise PPS rotation. For example, the control points (i,k)∼(i,j) are selected and added to the control point set $S(i-1,k,l-\sum_{m=k}^{j}L_{i,m})$ to meet the conditions of argmax(F(i,j,l)). Figure 3 shows the result of a SPArc_‐4pi_ optimization using the example of intracranial chordoma cancer.

#### 2.1.3 Control points re‐sampling and fine‐tuned optimization

Once the SPArc_‐4pi_ optimization and trajectory search (2.1.2) are completed, the plan will then be re‐sampled to a 2.5‐deg sampling frequency along the selected SPArc_‐4pi_ trajectory based on the energy layer delivery sequence.^1^ The effective ± 1.25 degree tolerance window can ensure the treatment delivery accuracy, mitigating the difference between the nominal plan and the delivered plan.^41^

### 2.2 Patient selection and plan generation

#### 2.2.1 Patient selection

Five representative clinical cases were retrospectively selected in this study, including two intracranial target cases, one bilateral HN case, one lung and one pancreatic case. Table 1 includes detailed information and prescription doses for the selected cases, and the Institutional Review Board approved the study (2017‐455).

**TABLE 1:** Target volume and dosimetric parameters for IMPT, SPArc_‐2d_, and SPArc_‐4pi._

Tumor site	CTV #	CTV(cc)	Prescription Dose(cGy)
Bilateral HN	1	93.50	7000
(Supraglottis)			
	2	273.57	5400
Partial Brain	1	221.89	4500
Clival Chordoma	1	16.17	7020
	2	32.67	5040
Lung	1	123.54	6000
Pancreatic	1	290.07	3000
	2	64.80	4500

#### 2.2.2 Plan generation

For each case, three different plans were generated: (1) IMPT, (2) SPArc_‐2d_, and (3) SPArc_‐4pi_. The same robust optimization settings were used, such as setup uncertainty of ± 2 to ± 5 mm depending on disease sites and range uncertainty of ± 3.5%. The IMPT plans used clinical beam angles. The SPArc_‐2d_ plans are generated based on the previously published optimization algorithm implemented in the RayStation via in‐house scripting^21^ via a single co‐planar arc and 2.5 deg sampling frequency. SPArc_‐4pi_ were generated based on the optimization algorithm described in section 2.1 and implemented in the RayStation via in‐house scripting. Detailed plan parameters, such as the number of fields, arc trajectories, and range, were listed in Table 2. All the plans utilized the same optimization objectives and constraints.

**TABLE 2:** Plan generation information for IMPT, SPArc_‐2d_ and SPArc_‐4pi_ plans, EL stands for energy layer.

Tumor site	IMPT		SPArc‐2d		SPArc‐4pi		
	Fields#	EL #	Arc range	EL#	Arc range	EL#	f
Bilateral HN	4(G30,T0)	192	Full Arc	144	4pi Arc	128	0.3
	(G120,T0)		(G0∼360,T0)		(G30∼160,		
	(G240,T0)				T180∼330),280		
	(G330,T0)						
Partial Brain	2(G160,T180)	68	Full Arc	142	4pi Arc	192	0.3
	(G186,T180)		(G0∼360,T0)		(G80∼160,		
					T180∼330),480		
Clival Chordoma	3(G0,T0)	77	Full Arc	142	4pi Arc	106	0.4
	(G120,T0)		(G0∼360,T0)		(G100∼160,		
	(G240,T0)				T180∼320),265		
Lung	2(G180,T0)	46	Partial Arc	182	4pi Arc	149	0.5
	(G240,T0)		G151.25∼23.75,T0		(G11.25∼160,		
					T176.25∼240),370		
Pancreatic	2(G90,T0)	56	Partial Arc	80	4pi Arc	169	0.4
	(G160,T0)		G61.25∼258.75,T0		(G20∼160,		
					T176.25∼320),430		

### 2.3 Dosimetry comparison

The plan quality was compared among IMPT, SPArc_‐2d_, and SPArc_‐4pi_ planning groups, including the target conformity index (CI), which is defined as:

(3) $$CI=\frac{target\;volume\;covered\;by\;isodose}{the\;total\;isodose\;volume}$$

Dose‐volume histograms (DVHs) of the target and dosimetric metrics of organs at risk (OARs) were compared as well, including V98 for CTVs, maximum point dose, mean dose .etc. for OARs. Additionally, the integral dose (ID) is also evaluated, which is defined as:

(4) $$ID\left(Gy*L\right)=D\left(Gy\right)*V\left(L\right)$$

where D is the mean dose delivered to total volume V.

### 2.4 Delivery time comparison

The treatment delivery time is simulated based on a published and validated proton machine‐specific delivery sequence model.^38^ Two kinds of beam delivery time were used for comparison purposes: (a) static beam delivery time (BDT*_s_*) and (b) dynamic beam delivery time (BDT*_d_*). BDT*_s_* are simulated by delivering the treatment plan via a fixed gantry angle which includes the spot spill time, spot scan time, and energy layer switch time without considering the gantry or PPS rotation or their mechanical limitation. The BDT_d_ includes both static irradiation time (BDT*_s_*) and mechanical rotation time. The mechanical rotation time includes the PPS and gantry movement based on the mechanical constraints of 6 deg/s maximum speed and 0.6 deg/s^2^ maximum acceleration or deceleration. For the IMPT plan, the gantry and PPS movement are simulated in a similar manner without considering the field transfer time between the Oncology Information System, e.g., Mosaiq or ARIA, to the Proton Therapy System.

## 3 RESULT

### 3.1 Plan quality comparison

**FIGURE 4:**
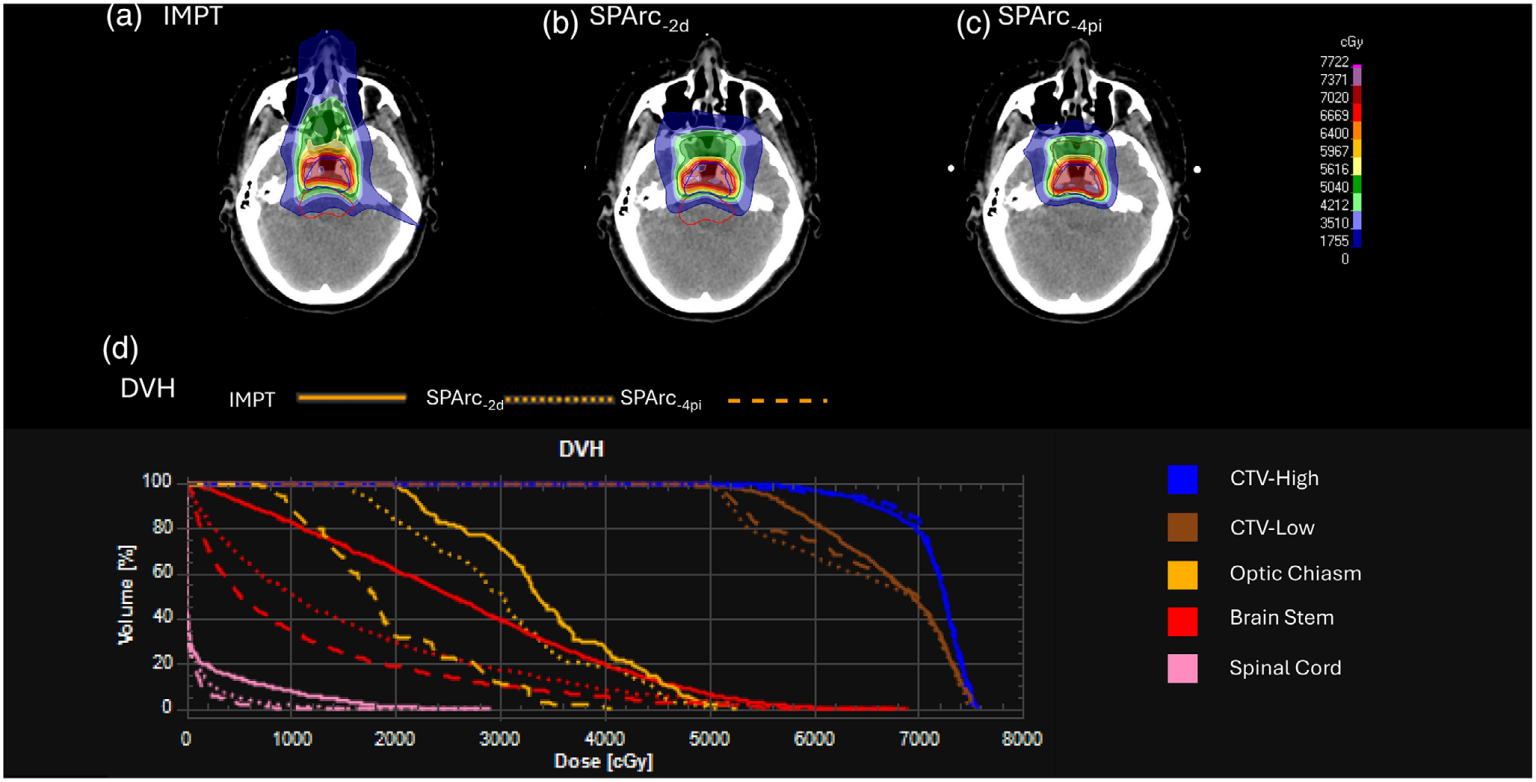
The plan quality and DVH comparison among IMPT, SPArc_‐2d_, and SPArc_‐4pi_ plan for Clival Chordoma cancer. (A) IMPT; (B) SPArc_‐2d_; (C) SPArc_‐4pi_; (D) DVH shows the dosimetric difference among IMPT, SPArc_‐2d_, and SPArc_‐4pi_.

**FIGURE 5:**
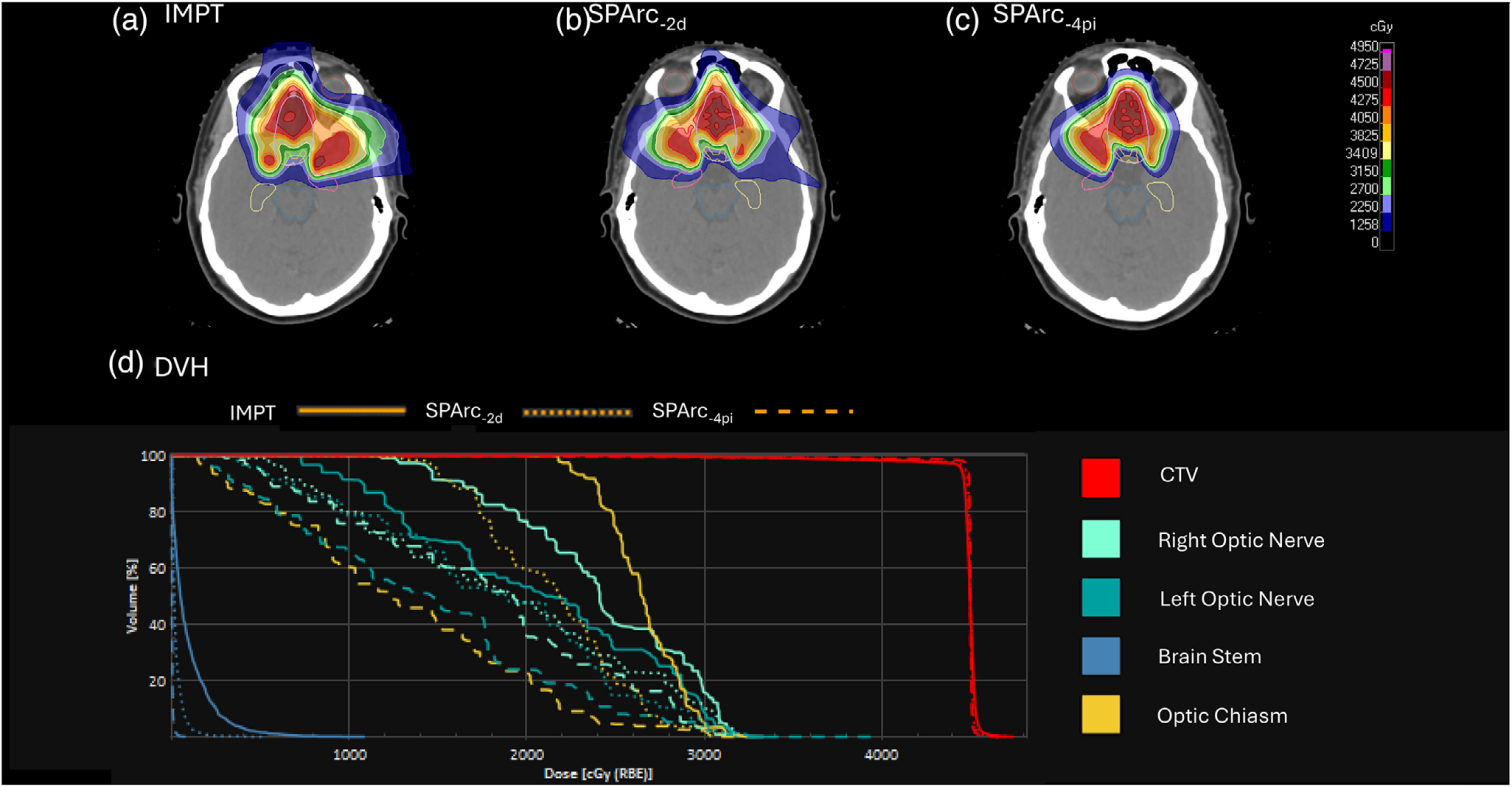
The plan quality and DVH comparison among IMPT, SPArc‐2d, and SPArc‐4pi plans for Partial Brain cancer. (A) IMPT; (B) SPArc_‐2d_; (C) SPArc_‐4pi_; (D) DVH shows the dosimetric difference among IMPT, SPArc_‐2d_, and SPArc_‐4pi_.

**FIGURE 6:**
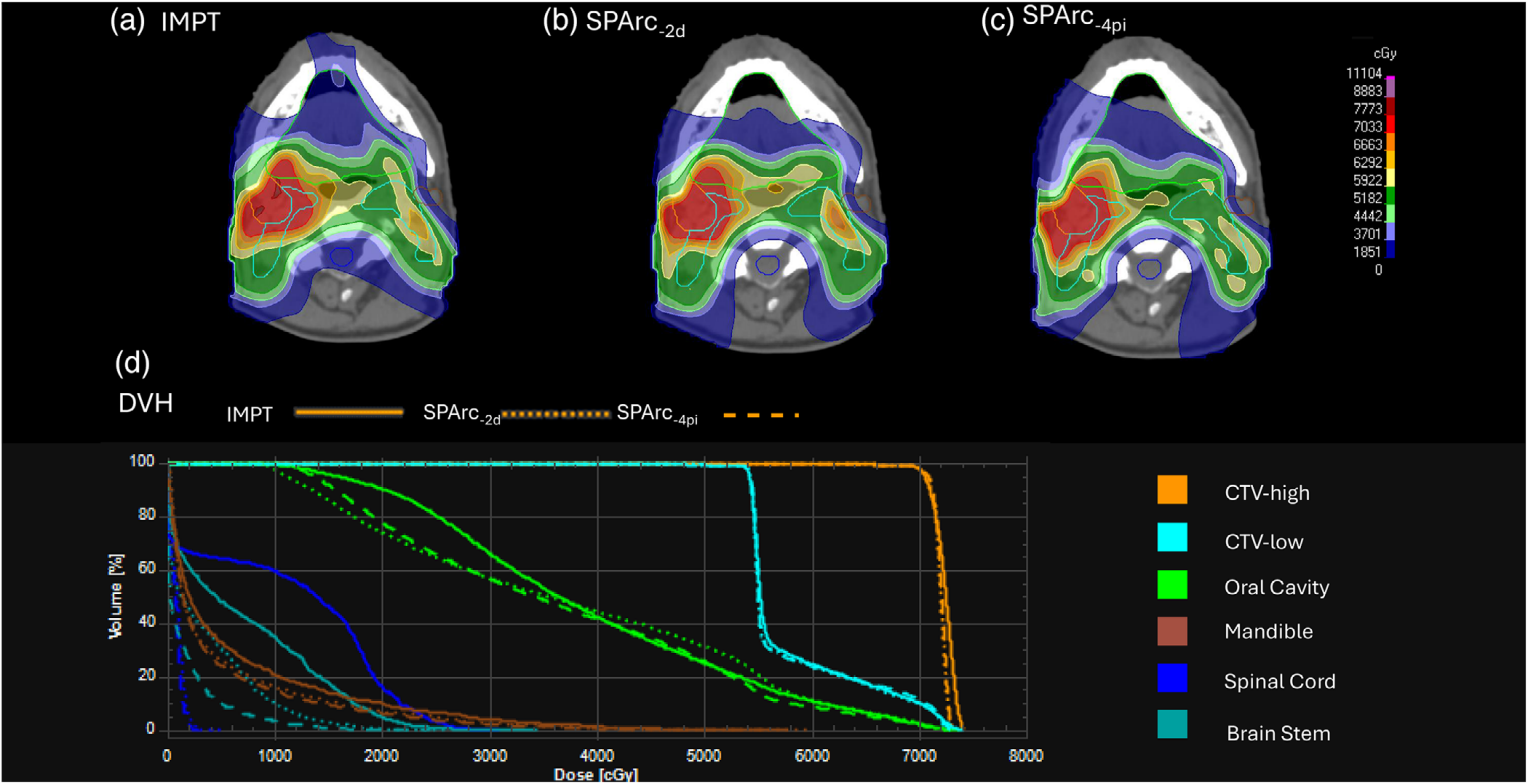
The plan quality and DVH comparison among IMPT, SPArc_‐2d_, and SPArc_‐4pi_ plans for Bilateral HN cancer. (A) IMPT; (B) SPArc_‐2d_; (C) SPArc_‐4pi_; (D) DVH shows the dosimetric difference among IMPT, SPArc_‐2d_, and SPArc_‐4pi_.

**FIGURE 7:**
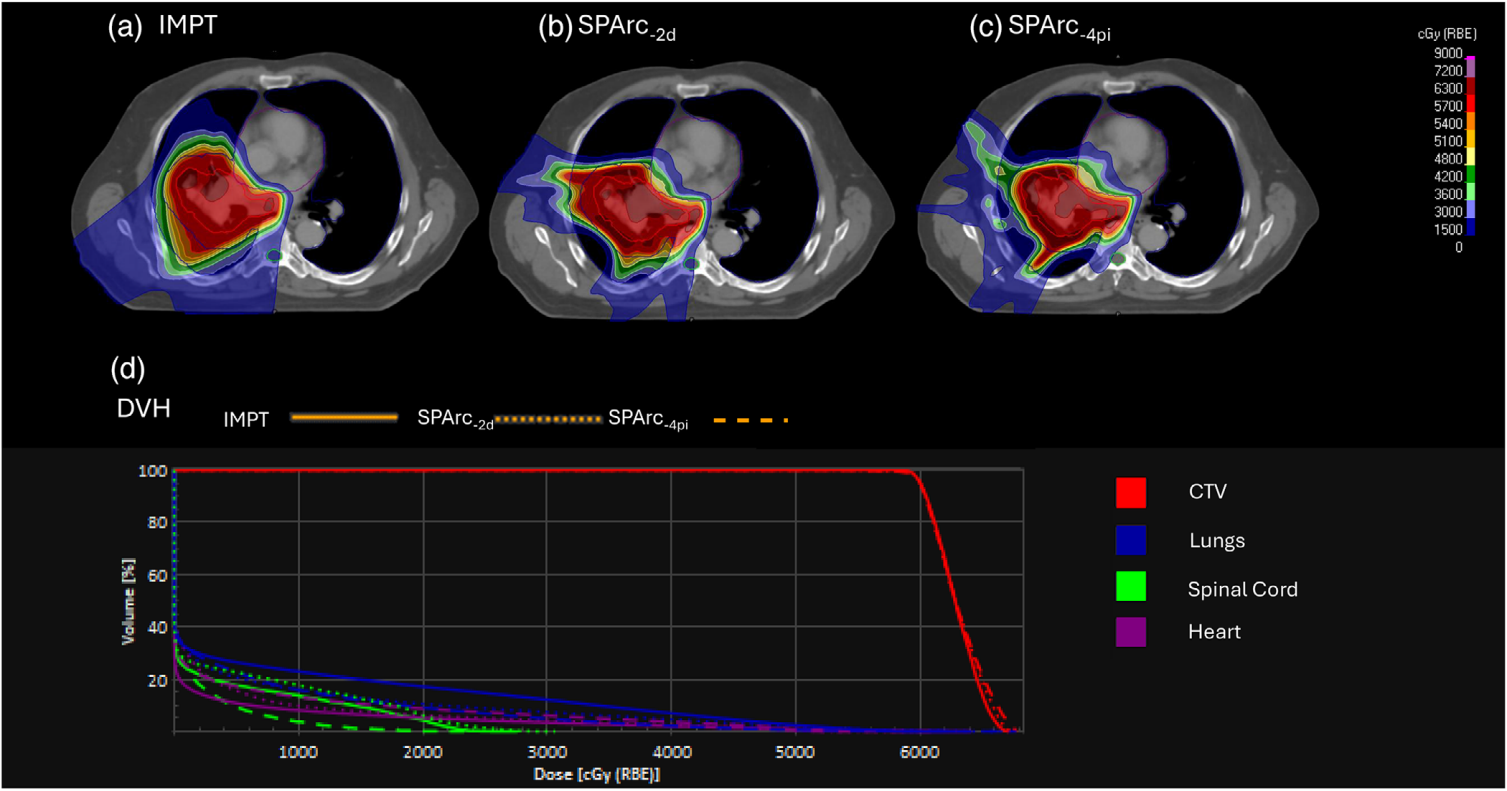
The plan quality and DVH comparison among IMPT, SPArc_‐2d_, and SPArc_‐4pi_ plans for Lung cancer. (A) IMPT; (B) SPArc_‐2d_; (C) SPArc_‐4pi_; (D) DVH shows the dosimetric difference among IMPT, SPArc_‐2d_, and SPArc_‐4pi_.

**FIGURE 8:**
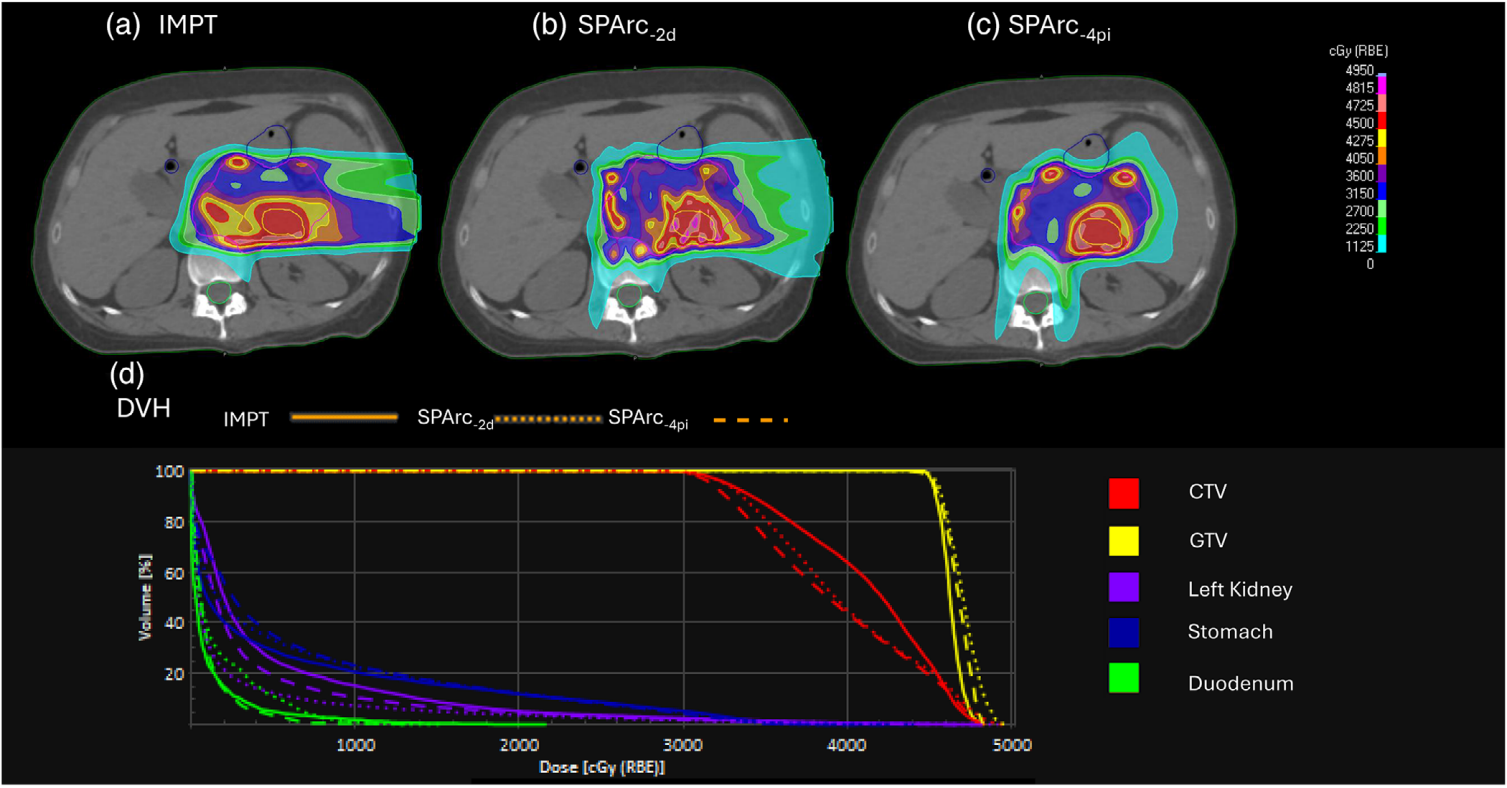
The plan quality and DVH comparison among IMPT, SPArc_‐2d_, and SPArc_‐4pi_ plans for Pancreatic cancer. (A) IMPT; (B) SPArc_‐2d_; (C) SPArc_‐4pi_; (D) DVH shows the dosimetric difference among IMPT, SPArc_‐2d_, and SPArc_‐4pi_.

Plan quality and DVH comparisons are shown in Table 3 and Figures 4, 5, 6, 7 and 8. In general, the SPArc_‐4pi_ outperforms IMPT and SPArc_‐2d_ in terms of plan quality, including OARs sparing(e.g. Spinal Cord, IMPT: 18.18 ± 8.74 Gy, SPArc_‐2d_:11.78 ± 9.90 Gy, SPArc_‐4pi_:9.29 ± 7.36 Gy, *P* = 0.05 for IMPT, *P* = 0.05 for SPArc_‐2d_), dose conformity to the target(IMPT: 0.47 ± 0.15, SPArc_‐2d_:0.54 ± 0.20, SPArc_‐4pi_:0.60 ± 0.20, *P* < 0.01 for both IMPT and SPArc_‐2d_), and reduced ID for IMPT(IMPT: 57.53 ± 38.43 Gy*L, SPArc_‐2d_:55.06 ± 37.57 Gy*L, SPArc_‐4pi_:54.20 ± 38.02 Gy*L, *P* = 0.11 for IMPT, *P* = 0.30 for SPArc_‐2d_).

**TABLE 3:** Dosimetric comparison among IMPT, SPArc‐2d and SPArc‐4pi.

Structure	Value	IMPT	SPArc_‐2d_	SPArc_‐4pi_
**Clival Chordoma**				
CTV‐High(7020cGy)	V100(%)	78.75	80.37	82.27
	CI	0.52	0.61	0.61
CTV‐Low(5040cGy)	D98(Gy)	52.94	50.67	50.68
	CI	0.33	0.43	0.52
Brain Stem	Max(Gy)	58.65	55.58	53.41
	Mean(Gy)	26.08	15.31	10.62
Spinal Cord	Max(Gy)	19.73	11.28	7.45
Optic Chiasm	Max(Gy)	52.02	49.46	40.33
	Mean(Gy)	34.33	30.23	19.61
Left Optic Nerve	Max(Gy)	50.45	48.15	47.46
Right Optic Nerve	Max(Gy)	46.47	45.34	42.53
Left Eye	Max(Gy)	14.37	7.05	0.73
Right Eye	Max(Gy)	8.19	7.01	7.08
Left Cochlea	Max(Gy)	16.21	12.97	12.13
Right Cochlea	Max(Gy)	17.33	16.43	15.52
Oral Cavity	Max(Gy)	14.21	4.86	1.30
	Mean(Gy)	1.07	0.39	0.06
Integral Dose	(Gy*L)	21.56	18.84	15.14
**Partial Brain**				
CTV	V98(%)	97.06	97.46	98.47
	CI	0.69	0.86	0.90
Brain Stem	Max(Gy)	6.07	1.69	0.30
Left Eye	Max(Gy)	14.37	5.52	3.20
Right Eye	Max(Gy)	16.59	7.67	4.87
Left Hippocampus	Max(Gy)	16.67	13.28	7.98
Right Hippocampus	Max(Gy)	35.03	30.25	27.17
Optic Chiasm	Max(Gy)	31.25	30.84	30.18
	Mean(Gy)	26.52	21.44	13.06
Left Optic Nerve	Max(Gy)	31.88	31.52	31.50
	Mean(Gy)	20.45	17.95	14.23
Right Optic Nerve	Max(Gy)	31.75	31.70	30.60
	Mean(Gy)	24.03	18.62	17.60
Integral Dose	(Gy*L)	39.62	36.30	32.30
**Bilateral HN**				
CTV‐high	V98(%)	99.05	99.08	99.04
	CI	0.37	0.39	0.40
CTV‐low	V98(%)	99.03	99.16	99.18
	CI	0.32	0.35	0.36
Brain Stem	Max(Gy)	24.38	17.89	14.35
	Mean(Gy)	7.09	3.58	1.70
Spinal Cord	Max(Gy)	26.84	2.75	2.18
Oral Cavity	Max(Gy)	71.35	71.40	71.19
Parotid	Mean(Gy)	6.33	5.13	4.66
Mandible	Max(Gy)	53.41	53.71	49.20
Integral Dose	(Gy*L)	117.94	114.59	112.83
**Lung**				
CTV	D95	60.00	60.00	60.00
	CI	0.40	0.40	0.49
Lungs	V20Gy(%)	17.14	10.54	9.16
	V5Gy(%)	26.46	20.67	22.68
	Mean(Gy)	7.67	5.42	4.76
Esophagus	Mean(Gy)	0.95	1.92	2.10
Heart	Mean(Gy)	2.70	3.55	4.36
Spinal Cord	Max(Gy)	25.52	28.55	21.66
Integral dose	(Gy*L)	71.92	67.38	68.33
**Pancreatic**				
CTV	D98(Gy)	31.39	31.37	30.77
	CI	0.54	0.62	0.69
GTV	D98(Gy)	45.00	45.00	45.00
Left Kidney	Mean(Gy)	4.92	2.73	3.88
Spinal Cord	Max(Gy)	6.22	6.91	6.21
Duodenum	Max(Gy)	9.39	8.44	6.08
Stomach	Max(Gy)	35.14	34.75	34.56
Bowel	Max(Gy)	38.98	39.81	38.56
Integral dose	(Gy*L)	36.63	38.17	42.39

#### 3.1.1 Clival chordoma

The SPArc_‐4pi_ showed improved dosimetric plan quality over IMPT and SPArc_‐2d_ and improved the CI from 0.52, 0.33 (IMPT) and 0.61, 0.43 (SPArc_‐2d_) to 0.61,0.52(SPArc_‐4pi_). More specifically, SPArc_‐4pi_ reduced the maximum point dose of optical chiasm to 40.33 Gy compared to IMPT (52.02 Gy, 22.47% reduction) and SPArc_‐2d_(49.46 Gy, 18.45% reduction). SPArc_‐4pi_ also reduced the mean dose to the brain stem from 26.08 Gy (IMPT) and 15.31 Gy (SPArc_‐2d_) to 10.62G y (SPArc_‐4pi_), respectively. Furthermore, SPArc_‐4pi_ reduced the maximum and mean dose to the oral cavity from 14.21 Gy, 1.07 Gy (IMPT) and 4.86 Gy, 0.39 Gy (SPArc_‐2d_) to 1.30 Gy and 0.06 Gy, respectively. Most interestingly, even with more degree of freedom and beam angles, SPArc_‐4pi_ is able to reduce the ID by 30.87% (IMPT), and 18.93% (SPArc_‐2d_).

#### 3.1.2 Paritial brain

The SPArc_‐4pi_ showed improved dosimetric plan quality over IMPT and SPArc_‐2d_ and improved the CI from 0.69 (IMPT) and 0.86 (SPArc_‐4pi_) to 0.90. More specifically, SPArc_‐4pi_ reduced the maximum point dose of the left hippocampus to 7.98 Gy compared to IMPT (16.67 Gy, 52.13% reduction) and SPArc_‐2d_(13.28 Gy, 39.91% reduction). SPArc_‐4pi_ also reduced the mean dose to the optical chiasm from 26.52 Gy (IMPT) and 21.44 Gy (SPArc_‐2d_) to 13.06G y (SPArc_‐4pi_), respectively. Furthermore, SPArc_‐4pi_ significantly reduced the maximum dose to the left eye from 14.37 Gy (IMPT) and 5.52 Gy (SPArc_‐2d_) to 3.20 Gy and a similar reduction is witnessed for the right eye. SPArc_‐4pi_ is able to reduce the ID by 18.48% (IMPT), and 11.00% (SPArc_‐2d_).

#### 3.1.3 Bilateral HN (Supraglottis)

The SPArc_‐4pi_ showed improved dosimetric plan quality over IMPT and SPArc_‐2d_ and achieved slightly improved CI of 0.40 compared to 0.37 (IMPT) and 0.39 (SPArc_‐2d_). More specifically, SPArc_‐4pi_ reduced the maximum point dose of the brain stem to 14.35 Gy compared to IMPT (24.38 Gy, 41.14% reduction) and SPArc_‐2d_(17.89 Gy, 19.79% reduction). SPArc_‐4pi_ also reduced the maximum point dose to the spinal cord from 26.84 Gy (IMPT) and 2.75 Gy (SPArc_‐2d_) to 2.18G y (SPArc_‐4pi_), respectively. In addition, SPArc_‐4pi_ is able to slightly reduce the ID by 4.33% (IMPT), and 1.54% (SPArc_‐2d_).

#### 3.1.4 Lung cancer

The SPArc_‐4pi_ showed improved dosimetric plan quality over IMPT and SPArc_‐2d_ and achieved an improved CI of 0.49 compared to 0.40 (IMPT) and 0.40 (SPArc_‐2d_). More specifically, SPArc_‐4pi_ reduced the maximum point dose of the spinal cord to 21.66 Gy compared to IMPT (25.52 Gy, 15.13% reduction) and SPArc_‐2d_(28.55 Gy, 24.13% reduction). SPArc_‐4pi_ also reduced the mean dose to the lungs from 7.67 Gy (IMPT) and 5.42 Gy (SPArc_‐2d_) to 4.76G y (SPArc_‐4pi_), respectively. The V20Gy of lungs is reduced from 17.14% (IMPT and 10.54%(SPArc_‐2d_) to 9.16% (SPArc_‐4pi_).

#### 3.1.5 Pancreatic cancer

The SPArc_‐4pi_ showed improved dosimetric plan quality over IMPT and SPArc_‐2d_ and achieved an improved CI of 0.69 compared to 0.54 (IMPT) and 0.62 (SPArc_‐2d_). More specifically, SPArc_‐4pi_ reduced the maximum point dose of the duodenum to 6.08 Gy compared to IMPT (9.39 Gy, 35.25% reduction) and SPArc_‐2d_ (8.44 Gy, 27.96% reduction). SPArc_‐4pi_ also reduced the mean dose to the left kidney from 4.92 Gy (IMPT) to 3.88G y (SPArc_‐4pi_) but slightly increased compared to SPArc_‐2d_ (2.73 Gy), respectively.

### 3.2 Delivery time comparison

Table 4 includes the comparison of the delivery time between IMPT, SPArc_‐2d_, and SPArc_‐4pi_ plans. The SPArc_‐4pi_ plans show no significant delivery time cost increase compared to SPArc overall. Noting that the IMPT plan will introduce set‐up time, taking minutes to complete for each control point, which is not included in this result, the SPArc_‐4pi_ can outperform IMPT plans in clinical practice.

**TABLE 4:** Static and dynamic delivery time comparison among IMPT, SPArc_‐2d_, and SPArc_‐4pi_ plans.

	IMPT	SPArc_‐2d_	SPArc_‐4pi_
**Clival Chordoma**			
Irradiation time(sec)	76.23	92.69	98.77
Energy layer switch time(sec)	66.10	158.50	199.70
Total Static time(sec)	127.33	251.19	298.47
Total Dynamic time(sec)	147.33	307.48	349.88
**Partial Brain**			
Irradiation time(sec)	129.71	102.37	113.96
Energy layer switch time(sec)	56.20	271.94	348.11
Total Static time(sec)	175.91	374.31	462.07
Total Dynamic time(sec)	185.21	378.64	465.15
**Bilateral HN**			
Irradiation time(sec)	349.86	437.74	436.16
Energy layer switch time(sec)	151.60	173.20	153.40
Total Static time(sec)	481.46	610.94	589.56
Total Dynamic time(sec)	536.10	668.10	644.12
**Lung**			
Irradiation time(sec)	90.14	182.99	122.47
Energy layer switch time(sec)	30.80	174.00	168.10
Total Static time(sec)	120.94	356.99	290.57
Total Dynamic time(sec)	137.77	411.66	329.68
**Pancreatic**			
Irradiation time(sec)	123.49	265.06	208.65
Energy layer switch time(sec)	42.8	94.00	156.18
Total Static time(sec)	166.29	359.06	364.83
Total Dynamic time(sec)	179.73	362.66	384.30

## 4 DISCUSSION

This is the first study to investigate the feasibility of utilizing a greater degree of freedom in plan optimization and treatment delivery in particle beam therapy. The SPArc_‐4pi_ optimization framework is derived from solutions to the classic 0–1 knapsack problem, which is to select an optimized subset from a set of objects with different costs and values with a total cost limitation in typical DP algorithms,^42^ adapted to operate within the 4pi spatial domain to address the treatment planning and delivery requirements from the spherical surface geometry. This study incorporates DP into the SPArc_‐4pi_ optimization algorithm to search for the optimal plan quality and trajectory for efficient treatment delivery and then benchmarked it with conventional multi‐field IMPT and state‐of‐the‐art SPArc_‐2d_ plans.

These exciting results demonstrated three key points (1) Optimization across the entire 4pi space has the potential for a better plan quality compared to the conventional multifield‐IMPT and SPArc_‐2d_ therapy. This new finding opens a new opportunity to advance the technology for particle beam therapy to the 4pi space. (2) The SPArc_‐4pi_ plan, a mathematical challenge problem with numerous possible solutions such as energy layers, spots, and trajectories, could be solved through a dynamic programming approach within a reasonable time based on a standard office workstation. The DP optimization costs less than 10 seconds for all 5 cases included, with workstation specifications of Intel(R) Core(TM) i5‐12500T 2.00 GHz, 16GB memory, no GPU. After that, each plan will need resampling and spot fine‐tuning, which is illustrated by Raystation 2023B, with a general time cost of 20 minutes. Additionally, (3) SPArc_‐4pi_ plan can be delivered within a reasonable treatment time compared to the IMPT and SPArc_‐2d_ plan based on the current proton therapy system's machine characteristics.^38^, ^43^

The new knowledge and approach developed from this study lay the cornerstone to further advance our particle beam therapy system to simultaneously irradiate the spot and switch energy layers while rotating the PPS and gantry movement for the optimal and efficient treatment plan for a broad range of disease sites and clinical indications. The representative case results demonstrate SPArc_‐4pi_’s potential to improve the dosimetric plan quality, especially for complicated patient geometry such as bilateral head neck cancer and intracranial targets abutting the optical nerves and brainstem. Additionally, SPArc_‐4pi_ is able to reduce the integral dose significantly compared to IMPT and SPArc_‐2d_ (Table 3). However, SPArc_‐4pi_ may increase the low dose volume compared to IMPT or SPArc_‐2d_ plan due to more entrance dose, which is similar to the finding from the comparable study between SPArc_‐2d_ and IMPT in previous work.^44^ The risk of secondary cancer in the pediatric patient population might be further investigated. Furthermore, we expect that the integration of SPArc_‐4pi_ with LET optimization will lead to a better capability in LET optimization and modulation because of the increased degree of freedom. This feature will be further developed along this direction.

Besides the dosimetric plan quality improvement, the trajectory search through SPArc_‐4pi_ optimization algorithm leads to a very reasonable treatment delivery time, contrary to the previous impression that it would introduce a tremendous time cost with numerous control points or beam angles via the entire 4pi space.^45^ The result showed that SPArc_‐4pi_ plan is comparable to a single 2D co‐planar arc SPArc_‐2d_ plan, with a better treatment plan quality. Please note that the treatment delivery time is based on the simulation, assuming the prototype SPArc_‐4pi_ system is ready for continuous irradiation while simultaneously rotating the PPS and gantry. More tests and experiments are needed to affirm these findings in the future, similar to the VMAT and SPArc techniques, once the proton therapy system is ready from an engineering perspective.^9^, ^38^

One of the limitations of the current SPArc_‐4pi_ algorithm is that it does not include collision avoidance trajectory search at this stage because treating head neck cancer or intracranial target using a 220‐degree partial gantry combined with robotic PPS has enough clearance for a collision‐free treatment delivery regardless of trajectory selection result. However, collision‐free trajectory search and delivery efficiency algorithms will be needed in the future if thoracic and abdomen targets start drawing significant clinical interest. Some of the particle gantry or treatment room designs might be less accessible compared to the IBA Proteus®ONE model. Thus, machine and treatment‐room‐specific collision models need to be developed in the future to fit various particle therapy systems and ensure safe and efficient clinical implementation. In addition, allowing the isocenter to move dynamically during treatment could offer further benefit for OAR sparing and collision avoidance, especially in large irradiation volume cases. This topic including potential dosimetric benefits and treatment delivery feasibility will be evaluated further.

This study represents an early exploratory investigation and efforts in this novel particle beam treatment delivery technique. It opens many topics for future development. For example, there will be opportunities for introducing a faster and more effective optimization algorithm to search for the control point and trajectories in the 4pi space, e.g., beam angle and sparsity optimization approach.^22^, ^26^ Another interesting topic that would be developed is the multi‐criteria optimization (MCO) platform to balance the treatment delivery time and plan quality so the clinician could quickly choose an optimal SPArc_‐4pi_ treatment plan from the Pareto‐surface.^24^ However, the problem of MCO for SPArc_‐4pi_ could be another mathematical challenge further down the road. With the optimization algorithm design from this study, it is possible to generate plans with different factor f, as a Pareto‐surface. Thus, the tradeoff could be explored between the plan quality SPArc_‐4pi_ plan and the number of total energy layers or trajectories. Additionally, it is also necessary to investigate the treatment delivery accuracy, tolerance window settings, and efficiency to ensure a precise clinical implementation similar to the VMAT and SPArc technique development.^46^

## 5 CONCLUSION

The study introduced the first SPArc_‐4pi_ algorithm for an efficient plan optimization and treatment delivery. The trajectory search and route decision‐making are achieved through a dynamic programming approach. This study not only shows SPArc_‐4pi_ ‘s potential to improve the plan quality via more degree of freedom compared to the conventional IMPT and state‐of‐art 2D co‐planar SPArc_‐2d_ technique but also demonstrates its feasibility for future clinical implementation based on the current proton beam therapy system's machine characteristics.

## CONFLICT OF INTEREST STATEMENT

Xuanfeng Ding received honorariums from IBA and Elekta speaker bureau outside the work presented here. Xuanfeng Ding and Xiaoqiang Li have a patent on Particle Arc therapy, which has been licensed to IBA, and was assigned to Corewell Health. There is a license agreement with IBA.
